# Surface Structure and Wetting Characteristics of Collembola Cuticles

**DOI:** 10.1371/journal.pone.0086783

**Published:** 2014-02-03

**Authors:** Håkon Gundersen, Hans Petter Leinaas, Christian Thaulow

**Affiliations:** 1 Department of Engineering Design and Materials, Norwegian University of Science and Technology (NTNU), Trondheim, Norway; 2 Department of Bioscience, University of Oslo (UIO), Oslo, Norway; Massachusetts Institute of Technology, United States of America

## Abstract

The cuticles of the arthropods Collembola (springtails) are known to be superhydrophobic, displaying such properties as water-repellence and plastron formation; overhanging surface structures have been suggested as the source of these properties. Superhydrophobicity is closely related to surface structuring and other surfaces with overhanging structures have been shown to possess robust superhydrophobic properties. In effort to correlate the wetting performance and surface structuring of the cuticles, from both a technical and evolutionary point of view, we investigated a selection of Collembola species including species from several families and covering habitats ranging from aquatic to very dry. The observed contact angles of wetting was in general larger than those predicted by the conventional models. Not all the studied Collembola were found to have superhydrophobic properties, indicating that superhydrophobicity is common, but not a universal trait in Collembola. Overhanging structures were found in some, but not all Collembola species with superhydrophobic cuticles; which leads to the conclusion that there is no direct link between overhanging surface structures and superhydrophobicity in Collembola.

## Introduction

Superhydrophobic surfaces, showing functional properties like self-cleaning, air-retention and drag reduction, have enjoyed increasing interest in recent years [1]–[4]. Evolution has led to a wide selection of different surfaces in nature, fitted for species in different environments. Some of these surfaces, such as the lotus leaf, the pitcher plant, and the cuticle and hairs of aquatic insects [5]–[11], have been the inspiration for biomimetic, superhydrophobic surfaces. Although excellent water shedding properties have been achieved the methods are often vulnerable to small imperfections or contamination, yield surfaces of low durability or are vulnerable to weathering [1], [12]–[14]. The investigation of natural surfaces that have yet to receive attention may provide inspiration for novel solutions to these challenges. The cuticles of Collembola (springtails) have long been known to be highly water-repellent [15]–[17]. Surface topography is know to be of vital importance to the wetting behavior of a surface, several studies have concluded that significant surface roughness is a prerequisite for superhydrophobic behavior [1], [2], [4], [8], [9]. Two recent studies have highlighted the cuticles of Collembola from a biomimetics point of view [18], [19]. One of these emphasized the importance of comparing species relatedness and habitat types in order to better understand the evolutionary aspects of the surface structure modifications [18]. The other study documented robust water shedding and air retention properties with water, as well as several organic liquids, on Collembola cuticles. Microscopic structures with overhang were suggested as a possible explanation for the superhydrophobic and omniphobic properties [19].

Superhydrophobicity is an effect that causes water to roll off a surface with very little resistance. This can result in a self-cleaning effect when contaminations adhere to the water droplets and roll off with the droplets. Upon submersion such surfaces can retain a thin layer of air on the surface; this greatly reduces flow resistance in water and is also the basis for the plastron respiration of arthropods [1], [8], [20], [21]. Generally a combination of a hydrophobic surface (displaying inherent contact angles (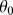) of 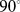 or more) and surface structuring is required to achieve superhydrophobic effects. Arthropod cuticles achieve this by combining cuticular structuring with a cover of tiny hairs and hydrophobic cuticular waxes [22].

**Figure 1:**
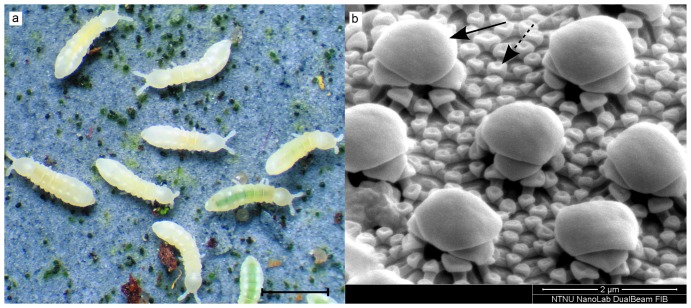
Live Collembola and their cuticle structure. (a) Several specimens of *Onychiurus sp.*, the scale bar is 1 mm. (b) SEM image showing the cuticle structure of *Onychiurus sp.*. A pattern of large, secondary granules (solid arrow) are shown and in between these a pattern of small, primary granules (dashed arrow), the primary granules are connected by ridges.

Collembola are small six-legged arthropods that represent one of the oldest and most abundant (in numbers of individuals) terrestrial animal groups on earth. Thus they have a long and diversified evolutionary history (nearly 400 million years) of adaptation to life on land. The cuticles of Collembola show complex surface structures, including both respiring surfaces and thicker parts that block gas exchange [15]. Figure 1 shows several Collembola and an SEM image of its cuticle. The cuticles are known to have strong anti-wetting properties, with the ability of plastron formation around submerged animals [17]. The variations in the patterns of surface structures, in relation to habitat conditions and species relatednes, is of both evolutionary and biomimetics interest. Collembola live on or below the ground and are thus highly affected by soil water conditions. Respiring through the body surface they also run the risk of dessication. In dryer habitats adaptation appears mainly to involve a reduction in the respiring surface and thereby an improved protection against water loss. Under wet conditions hydrophobic properties and improved plastron formation facilitating gas exchange may be more important [17]. Self-cleaning is also an important aspect of the superhydrophobic cuticle, as soil dwelling animals may come in intimate contact with harmful substances and pathogens. The appearance of the Collembola cuticles is often very clean, with no visible contamination even when studied in scanning electron microscopes (SEM).

The arthropod cuticle in general consists of a chitin-protein complex with a cover of epicuticular wax [22]. In Collembola, we find that thinner sections, without wax, and thicker parts covered by wax form recognizable geometric patterns. These thicker parts, or protuberances, are commonly referred to as granules. The basic pattern appears to consist of triangular granules connected by ridges in hexagonal rings [23]. The size of these basal units may vary, but are usually in the order of a few hundred nanometers. These granules have been shown to have overhanging structures in some species, where parts of the granule extend beyond the base of the granule, like the eaves of a roof. Helbig et al. suggested that this overhang was an important, but neglected, characteristic of non wetting Collembola cuticles[19]. Several triangular granules may fuse to quadrangular granules arranged in rectangular patterns. These structures, including both hexagonal and rectangular configurations, represent general patterns found in most Collembola at different parts of the body, and in all major taxonomic groups.

**Figure 2:**
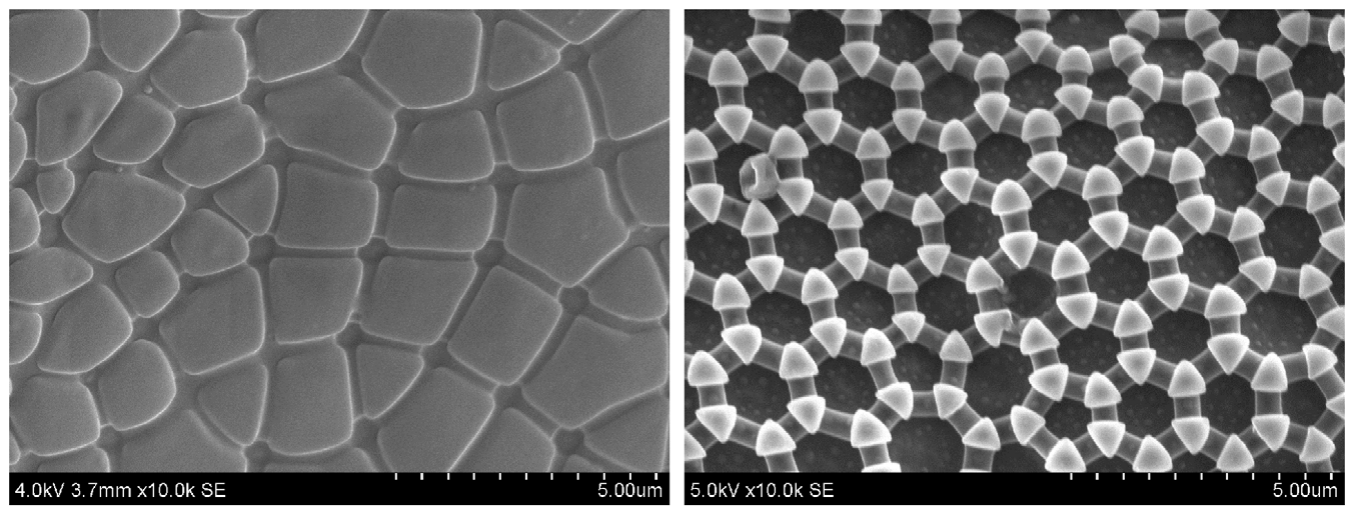
Scanning Electron Microscopy (SEM) images of species 10 and 12. Left: species 10 *A. laricis* Right: species 12 *X. maritima*. The images, at 10 000X magnification, show structures typical for the dorsal metasoma. Species 12 has a typical structure of triangular granules, connected by ridges, organized in a hexagonal pattern. Species 10 has markedly enlarged granules, in a variation of forms, organized in a varied pattern closer to square than hexagonal.

However, there are also some systematic differences between these groups, such as the tendency to form secondary granules (involving several primary granules) in several families of the superfamily (section) Poduromorpha. Figure 1 shows both larger, secondary granules and smaller, primary granules connected by ridges. In the superfamily Entomobryomorpha some genera of the family Isotomidae tend to modify their cuticle mainly by changing the connecting ridges and thus individual areas of thin cuticle between the granules, while in other genera the size of the individual granules may change greatly without affecting the size of the thin cuticle units. Lastly, other families of the Entomobryomorpha show little cuticle modification at all, possessing a more or less uniform cover of the hexagonal configuration, possibly with wax cover also on the thinner parts [16], [24]. Thus, in the latter group, it is possible that gas exchange occurs through the pores of the granules [16], rather than across the thinner parts of the cuticle, as argued for other Collembola groups [15], [25]. Figure 2 shows one species (*X. maritima*) with a typical hexagonal configuration and one species (*A. laricis*) with enlarged granules. Such systematic characteristics represent differences in the structural patterns on which evolution will act, and may lead to very different solutions to the same environmental challenges; i.e. between unrelated species living in the same habitat. This emphasizes that in order to improve our understanding of cuticular wetting properties from an evolutionary perspective, one should compare related species from different habitats as well as species from different families living in similar habitats.

In the present work we investigated the cuticles of twelves species of Collembola from four different Collembola families in an effort to explain the wetting properties of Collembola cuticles based on wetting principles and evolution. The selected species represent a wide range of habitats from high mountains to the coast, including both extreme drought as well as littoral and aquatic climes. Analyses of the structural arrangement of thinner and thicker parts of the cuticle, including the presence of overhang, were performed to quantify basic parameters in models of wetting behavior (e.g. roughness and solid surface fraction). The resulting, theoretical estimates of contact angles were compared with experimental contact angle measurements of water droplets on cuticles.

## Results

The advancing and receding contact angles of water droplets on the cuticles of a selection of Collembola was measured with the sessile drop technique, the contact angle hysteresis was calculated as the difference of the advancing and receding angles. The measured contact angles are presented in table 1; the uncertainty in the presented values correspond to one standard deviation as calculated from the population of measured values for each species.

**Table 1:** Contact Angle Measurement.

#	Species	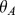	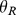	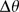	SG
1	*Hypogastura viatica*	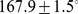	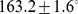	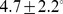	yes
2	*Isotomurus prasis*	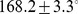	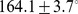	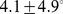	no
3	*Onychiurus sp.*	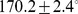	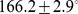	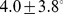	yes
4	*Folsomia quadrioculata*	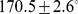	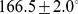	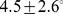	no
5	*Anurophorus septentrionalis*	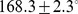	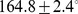	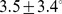	no
6	*Desoria olivacea*	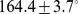	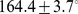	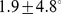	no
7	*Archisotoma besselsi*	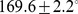	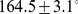	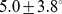	yes*
8	*Cryptopygus clavatus*	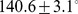	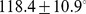	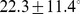	no
9	*Orchesella flavescens*	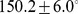	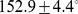	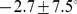	no
10	*Anurophorus laricis*	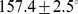	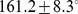	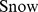	no
11	*Isotoma anglicana*	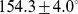	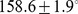	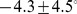	no
12	*Xenylla maritima*	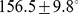	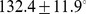	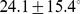	no

Results of the contact angle measurement for each of twelve species of Collembola. The measured advancing (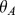) and receding (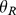) contact angles, whith standard deviation, the calculated contact angle hysteresis (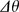) and the absence or presence of secondary granules (SG). * The secondary granules of *A. besselsi* are enlarged primary granules.

When the numbers in table 1 were compared to the standard criteria for superhydrophobicity (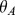 and 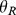 exceeding 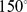 and 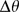 under 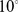 [3]) ten out of twelve tested species were found to be superhydrophobic. This includes species from all the tested families (Hypogasturidae, Onychiuridae, Isotomidae and Entomobrydiae) and a variety of habitats (intertidal zone, terrestrial, litter layer, watter-logged soil, forest floor, grassland and marsh) displaying the full range of moisture (very dry to aquatic) and flood danger (no danger to intertidal zone) [26]. Only species 8 and 12 (*C. clavatus* and *X. maritima*) were not superhydrophobic; water droplets were also observed to stick to these two species, where on all other tested species they would slide off. These two species represent two very different habitats; *C. clavatus* is active submerged in rock pools, and as such is always wet, while *X. maritima* lives on the crusts of lichens on boulders and standing tree trunks, which may become very dry for long periods [26].

**Figure 3:**
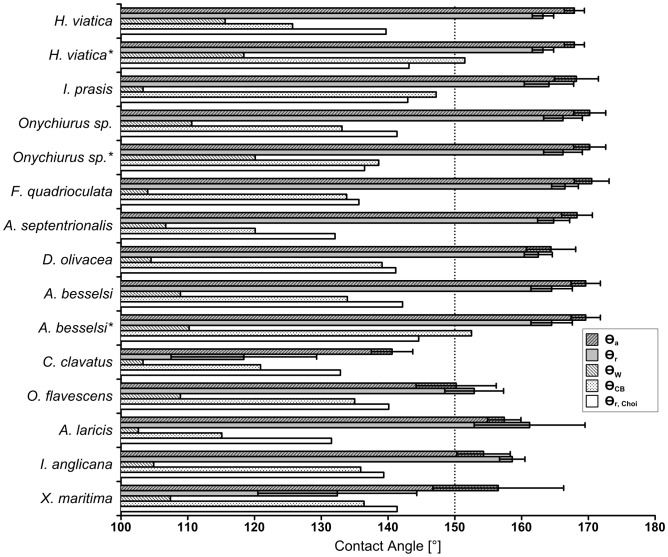
SEM images of FIB cross sections of species 10 and 12. Left: species 10 *A. laricis* magnification 8 000X, Right: species 12 *X. maritima* 15 000X magnification. The images show sections of the cuticle where a prism shaped part has been removed by FIB milling, while the structuring around it was protected by a layer of platinum, to reveal cross sections of the granules. A single granule is highlighted by a white circle in each image. In species 10 there is no evidence of overhang, in species 12 overhang is present.

Structural parameters were measured with scanning electron micrographs (SEM). The results are presented in table 2 where values for species marked with a * are based on secondary granules while the rest are based on primary granules. Figure 2 presents micrographs of two example species (species 10, *A. laricis* and species 12, *X. maritima*). Height data and the presence of overhang was determined from cross-sections created with focused ion beam (FIB) milling and subsequent SEM imaging. The FIB cross sections of *A. laricis* and *X. maritima* are shown in figure 3, note the presence of overhang on *X. maritima*. SEM images of the other species are included as supporting information, figures S1, S2, S3, and S4.

**Table 2:** Surface Structure Characteristics.

#	Species	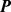 [nm]	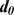 [nm]	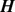 [nm]	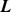 [nm]	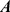 [ 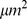 ]	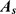 [ 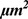 ]	Overhang
1	*H. viatica*	6	370	210	970	0.134	0.068	yes
1*	*H. viatica*	3	1950	1150	2790	3.66	0.54	yes
2	*I. prasis*	5	970	155	1570	0.77	0.15	no
3	*Onychiurus sp.*	6	380	175	750	0.108	0.041	yes
3*	*Onychiurus sp.*	3	1000	846	3180	2.55	0.77	yes
4	*F. quadrioculata*	4	860	290	2670	1.37	0.51	no
5	*A. septentrionalis*	3	570	332	4430	2.31	1.40	yes
6	*D. olivacea*	6	430	100	910	0.181	0.054	yes
7	*A. besselsi*	6	320	110	560	0.059	0.022	yes
7*	*A. besselsi*	4	620	355	1150	0.509	0.070	yes
8	*C. clavatus*	4	420	120	2240	0.655	0.39	no
9	*O. flavescens*	6	520	233	1230	0.278	0.098	no
10	*A. laricis*	4	490	165	5370	2.85	1.98	no
11	*I. anglicana*	4	630	193	1825	0.635	0.22	yes
12	*X. maritima*	6	1040	440	2350	1.06	0.35	yes

Surface structure characteristics, as measured on scanning electron micrographs. 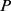: number of edges in the closest equivalent polygon; 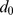: longest regular distance between primary granules; 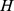: height of granules; 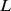: length of the three-phase contact line for the wetting system of one granule; 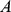: nominal area of a section of cuticle containing a single granule; 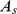 nominal surface area of a granule; the final column denotes the presence of overhanging structures. Rows marked * present values based on secondary granules.

**Figure 4:**
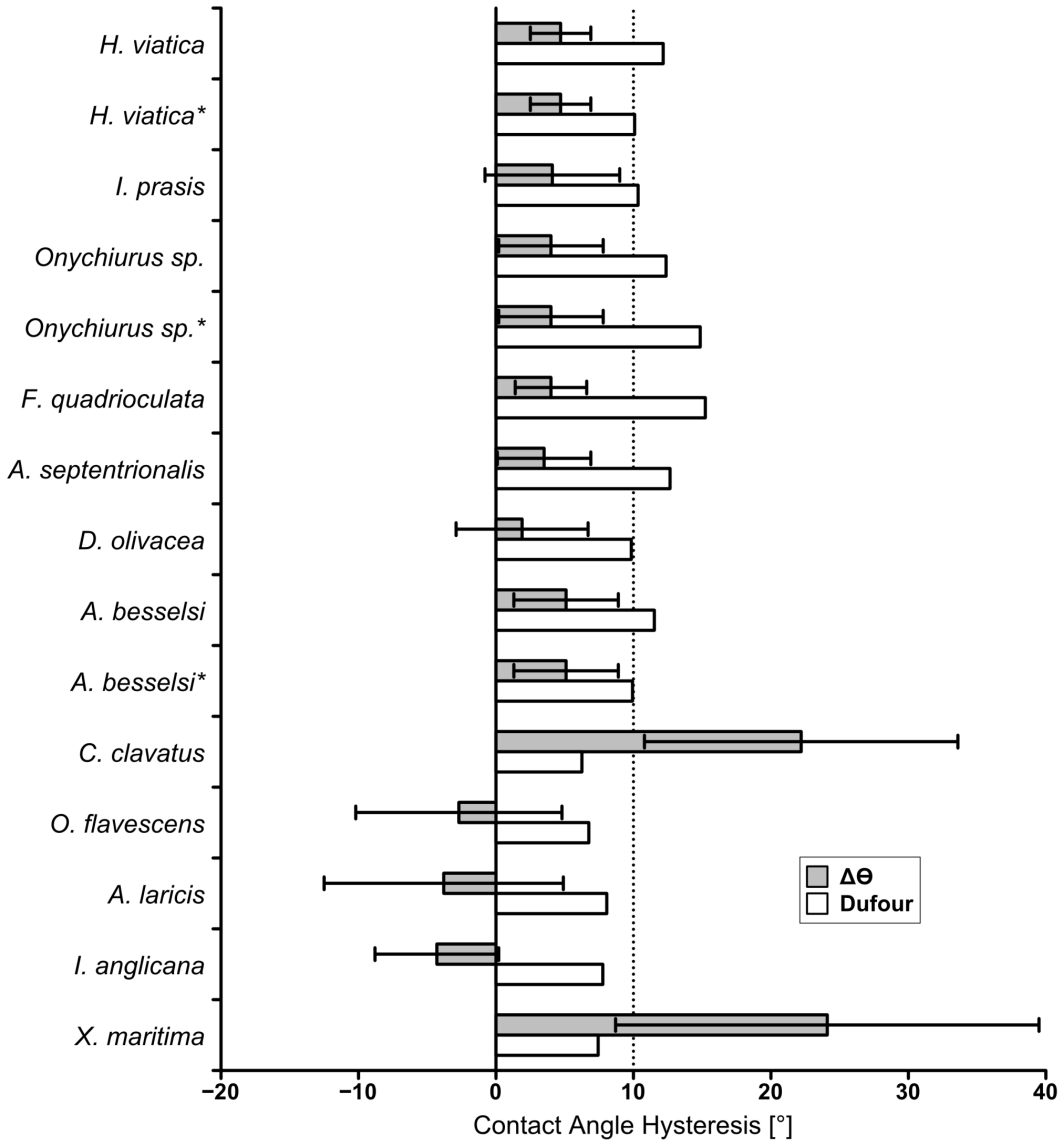
Measurement of structural parameters. The schematic shows how the structural parameters 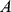, 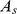, 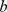, 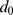, 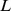 and 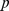 were measured from SEM images.

**Figure 5:**
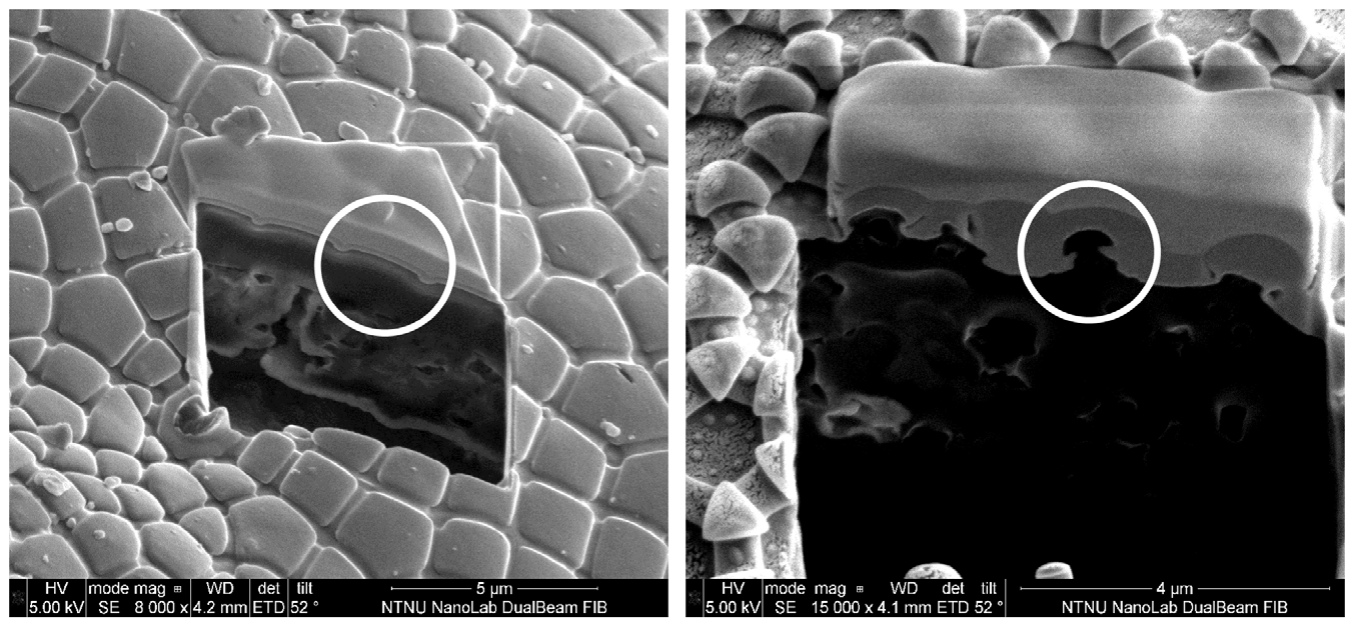
Measured contact angles compared to predicted contact angles. Measured advancing (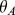) and receding (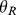) contact angles with one standard deviation error bars as compared to the values predicted by the Wenzel (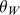), Cassie-Baxter (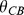) and the Choi (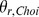) equations. The minimum limit for contact angles considered superhydrophobic is denoted by a dotted line at 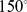. Rows marked with an asterisk (*) denote predicted values based on secondary granules.

**Figure 6:**
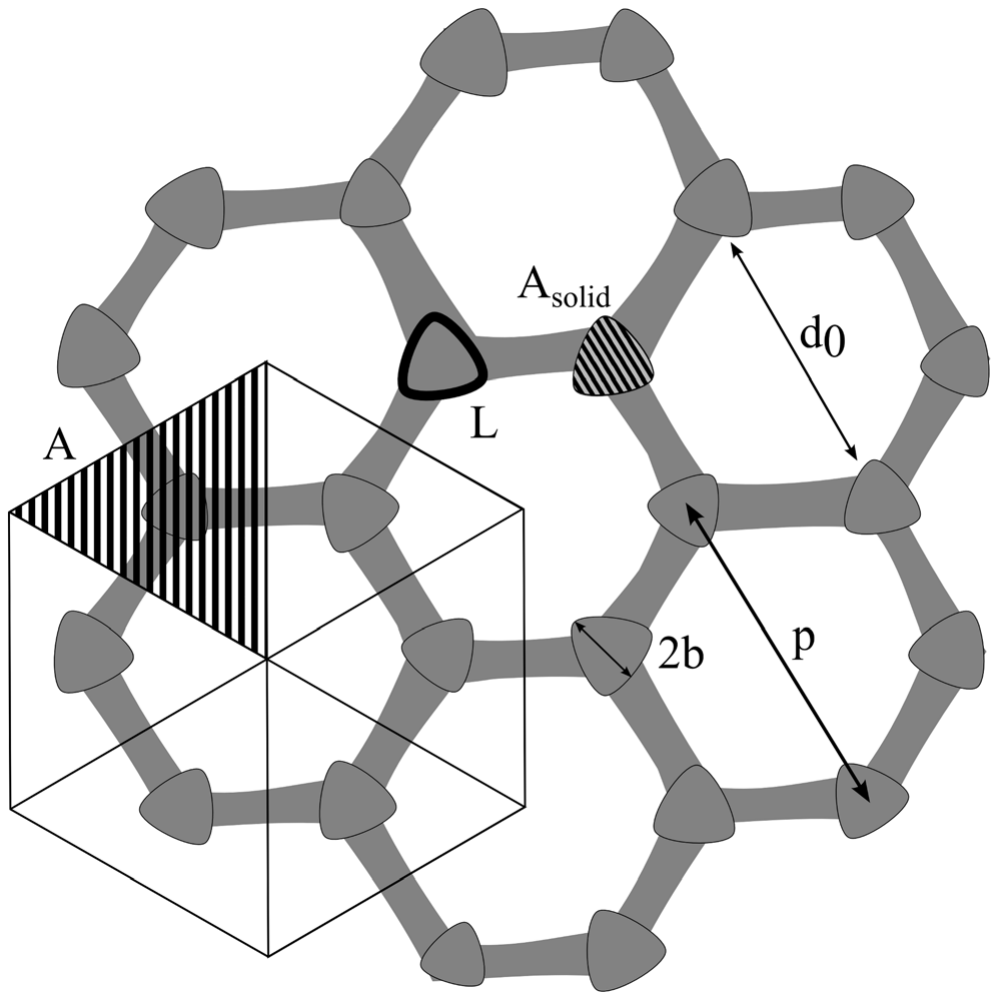
Measured contact angles hysteresis compared to contact angle hysteresis predicted by the Dufour method. The maximum limit for contact angle hysteresis considered superhydrophobic is denoted by a dotted line at 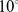. Rows marked with an asterisk (*) denote predicted values based on secondary granules.

Figure 4 illustrates how the different values were measured from cuticle micrographs. Based on these measurements (table 2) we estimated some important parameters (roughness factor (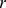), solid area fraction (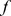), differential solid area fraction in the receding direction (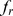), estimated contact angle from the Wenzel equation (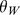) [27], estimated contact angle from the Cassie-Baxter equation (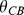) [29], estimated contact angle hysteresis from Dufour's method (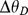) [30] and the estimated receding contact angle from Choi's method (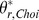) [30]) as presented in table 3. Neither the Wenzel equation, Dufour's method nor Choi's method predicted superhydrophobic behavior for any of the species. The Cassie-Baxter equation only predicted superhydrophobic behavior for species 1 and 7, and then only when the secondary granules were considered. The following assumptions were made: 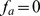, i.e. that the differential area fraction in the advancing direction is zero, which means that the advancing edge of a droplet is not in contact with the substrate. This assumption is reasonable for a system where the droplet is resting on the top of discrete asperities, since any incremental advancement of the contact line from a set of asperities will be into the empty space between asperities, where the solid area fraction is zero [30]. 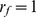, i.e. that the tops of the granules are assumed to be smooth. The inherent contact angle for the cuticle substrate was assumed to be 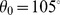. The predicted values are compared to the measured values in figure 5 for contact angles and in figure 6 for contact angle hysteresis; the figures are based on data from tables 1 and 3.

**Table 3:** Calculated Parameters.

#	Species	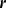	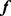	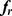	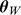	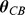	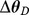	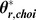
1	*H. viatica*	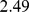	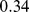	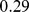	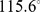	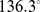	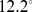	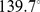
1*	*H. viatica*	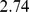	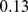	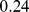	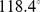	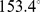	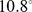	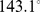
2	*I prasis*	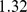	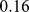	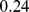	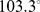	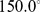	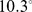	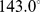
3	*Onychiurus sp.*	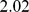	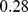	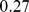	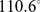	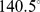	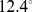	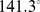
3*	*Onychiurus sp.*	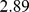	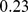	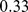	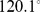	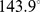	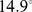	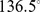
4	*F. quadrioculata*	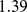	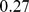	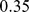	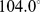	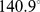	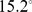	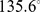
5	*A. septentrionalis*	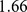	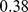	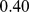	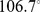	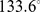	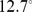	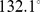
6	*D. olivacea*		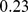	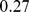	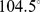	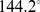	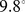	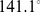
7	*A. besselsi*	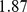	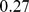	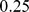	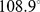	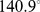	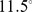	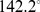
7*	*A. besselsi*	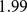	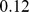	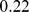	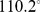	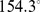	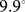	
8	*C. clavatus*	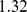	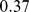	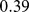	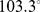	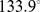		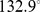
9	*O. flavescens*	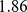	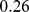	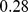	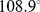	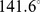	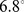	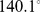
10	*A. laricis*	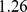	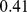	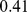	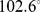	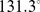	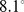	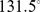
11	*I. anglicana*	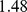	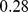	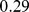	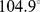	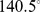	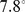	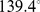
12	*X. maritima*	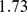	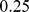	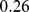	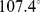	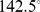	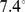	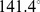

Calculated parameters, 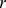: roughness factor, 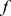: solid area fraction, 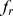: differential solid area fraction in the receding direction, 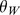: estimated contact angle from the Wenzel equation, 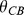: estimated contact angle from the Cassie-Baxter equation, 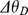: estimated contact angle hysteresis based on Dufour's method and 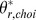: estimated receding contact angle based on Choi's method. Rows marked * present values based on secondary granules.

Wenzel's roughness parameter 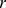 is the ratio between the nominal contact area and the actual contact area assuming complete wetting (i.e. Wenzel state); the different cuticular structures were approximated to repeating geometric patterns in order to estimate a value for 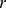. The solid fraction of the surface, 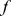, is used in the Cassie-Baxter relation, it can be estimated as 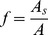, from the nominal area of the section of the cuticle containing a single granule (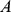) and the nominal surface area of the top of a granule (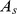). 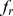 is the differential area fraction, as used by Choi et al. [30], found as 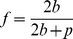 where the parameters 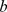 and 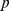 were measured from SEM images as illustrated in figure 4. 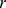, 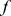, and 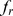 can, when combined with parameters from table 2 be used to calculate the contact angle for the surface as predicted by Wenzel's equation (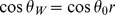) [27], the Cassie-Baxter equation (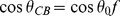) [28], Dufour's model for contact angle hysteresis (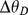) [29] and the receding contact angle according to Choi's model (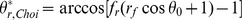) [30].

The structural parameters of the granules can also be estimated with nanoindenter atomic force microscopy (NI-AFM). This method proved more challenging than using SEM and FIB cross sections on Collembola cuticles and the primary granules of all species could not be imaged. Corresponding values based on NI-AFM are available for most of the species as supporting information, figures S5 and S6, tables S1 and S2.

## Discussion

The results presented in table 1 indicate that superhydrophobicity is a quite general characteristic of the Collembola cuticle, independent of habitat and phylogeny (relatedness). However, the two species *Xenylla maritima* and *Cryptopygus clavatus* showed some clear exceptions to this general trend.

As contact angles approach 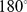 they get increasingly difficult to measure accurately [31]. The small size of the Collembola compared to the droplets used also makes accurate measurement challenging. The negative contact angle hystereses measured for several species are therefore likely the result of experimental error for samples with very small, but positive, contact angle hystereses. The qualitative observation of droplets sticking to two of the species (*Xenylla maritima* and *Cryptopygus clavatus*), however indicates significant contact angle hysteresis for these two species. Interestingly these two species occupy habitats at the two extreme ends of the humidity range. *C. clavatus* is active on the bottom of small rock pools [32]. A wettable cuticle seems to be a presupposition for the species to walk freely in a submerged state, as can be seen under stereo-microscope when they easily penetrate the water surface and become completely wetted (personal observation). *X. maritima*, in contrast, lives in drought exposed lichen crusts on rocks and tree trunks[26], where the risk of submersion is minimal. *X. maritima* shares this habitat with *A. laricis* which has a very different cuticle structure with distinct superhydrophobic properties. A possible explanation for these fundamentally different traits of two lichen living species is that they appear to have very different strategies to survive in their habitat. *A. laricis* is heavily protected against water loss by a heavy armor of large, wax-covered granules. While *X. maritima* is very tolerant to water loss (personal observations). *X. maritima* is quite inactive when dehydrated, and in order to utilize the improved conditions when humidity increases, it must recover the water balance quickly. A wettable cuticle may facilitate such recovery.

The contact angle experiments were only performed on the dorsal metasoma of the Collembola. This was because the droplets that could be produced in the experimental setup was relatively large compared to the size of the Collembola, such that placing a droplet on and examining the contact angle on smaller parts of the animal(e.g. antennas, limbs or head) was challenging and would not yield accurate or reproducible results. In short, the dorsal metasoma was the only area large enough and uniform enough to accommodate the measurement of contact angles. Only SEM and AFM images from the dorsal metasoma were used in the numerical analyses, as such the results of the contact angle measurements and the results of the mathematical predictions should be commensurable as they are both based solely on data from the dorsal metasoma.

There are normally three criteria that should be fulfilled for a surface to achieve a stable superhydrophobic effect: The intrinsic contact angle of the surface should be 90 or more (i.e. hydrophobic), surface structuring should create considerable roughness and the system should assume a Cassie-Baxter wetting state. Not all of the three criteria need necessarily be met, but each contribute to the stability of a superhydrophobic state [1], [8]. The epicuticular waxes of Collembola and other arthropods are known to be inherently hydrophobic, such that any wax-covered surface would contribute to water repellency [15], [16], [22]. Further, recent research into the effect of overhanging structures on wetting suggests that these structures may stabilize the Cassie-Baxter wetting state by providing a formidable energy barrier against wetting state transition [33]–[36]. The cuticles of several species of Collembola display overhang, which has been proposed as an explanation for the commonly observed excellent water repellency of Collembola cuticles by Helbig et al [19]. We must therefore consider the different surface structures, including overhang, of the studied Collembola to determine what creates the differences in wetting behavior.

In this study, we primarily emphasize the differences between the two non-superhydrophobic species (*X. maritima* and *C. clavatus*) and all other tested species. Though all tested species were hydrophobic (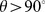) a considerable contact angle hysteresis was observed for these two species. A contact angle hysteresis in this range will hinder droplet movement on the cuticle surface, the result is that water droplets can stick to *X. maritima* and *C. clavatus*, albeit with a large contact angle, while they simply slide off the other tested species as the curvature of the cuticle itself is larger than the sliding angle of a droplet resting on the cuticle. This constitutes a clear, qualitative difference in the wetting behavior of these two species compared to the other tested species.

It is difficult to see any clear trends when we compare the structural parameters (table 2) between superhydrophobic and non-superhydrophobic species. *X. maritima* posesses the largest intergranular distance (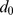) for primary granules, but this is not much larger than those of e.g. *I. prasis* and *F. quadrioculata*. *C. clavatus* has a rather small 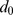. Another important parameter is the height of the granules (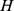). *X. maritima* had the highest primary granules of all investigated species, which should help to stabilize the Cassie-Baxter wetting state and enhance hydrophobic properties [37]. *C. clavatus* had the second smallest primary granule height. Thus, neither separate nor in combination did the two parameters discriminate between the species that did and did not show superhydrophobic properties. E.g. two of the superhydrophobic species *I. prasis* and *D. olivacea* had about the same 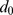 and smaller 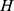 than *X. maritima* and *C. clavatus* respectively. Also, the contact line length (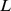) as well as the two area parameters (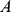 and 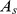) of both *X. maritima* and *C. clavatus* are midrange, neither exceptionally large nor small.

The roughness (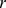) and solid area fraction (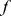) were calculated in order to evaluate the surfaces according to the theories of Wenzel, Cassie and Baxter [27], [28]. The results, shown in table 3 and compared with the measured values in figure 5, clearly underestimate the real contact angle of all studied species. The Collembola cuticles considered in this work are certainly more complex than the regular, geometric patterns considered by Wenzel, Cassie and Baxter. When describing such complex, natural surfaces with a single value for roughness 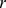 or area fraction 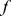, care was taken to make conservative translations. The values for 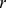 were calculated based on surface features smaller than those studied by Wenzel, while the values for 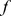 were calculated based on only the tops of the granules, thus neglecting the ridges; both of these considerations should lead to higher predicted contact angles for these models. The contact angle hysteresis was estimated based on Dufour's formula [29], but this overestimated the contact angle hystereses as compared to the measured values for all studied species, as shown in figure 6. Finally Choi's model [30] was used to estimate the receding contact angle, this underestimated the receding contact angle for all the superhydrophobic species, but interestingly overestimated it for *X. maritima* and *C. clavatus*.

Some key assumptions were made in the use of these models, namely that the intrinsic contact angle for the cuticle surface was 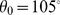, the tops of the asperities were smooth (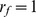) and the differential area fraction in the advancing direction was 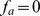. The intrinsic contact angle of waxes consisting mainly of hydrocarbon chains are expected to be in the range of 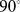 to 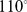, while the intrinsic contact angles of flat samples of the waxes of insects, as well as that of chitin are approximately 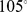 [22], [38]. If the intrinsic contact angles were larger than the assumed values, the predicted values of the Wenzel, Cassie-Baxter and Choi models would increase. Though even if the value was assumed to be as high as 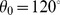 (a value typical for smooth polytetrafluoroethylene/Teflon) these models would only predict superhydrophobic behaviour for half of the investigated species. The models are still unable to differentiate between the superhydrophobic and non-superhydrophobic species, even with this unreasonably high estimated intrinsic contact angle. Smooth asperity tops is a less than likely assumption in that the granules display a slight curvature instead of completely flat plateaus, the assumption was made to simplify the Choi equation. The actual roughness parameter 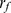 is likely between 1 and 2, as 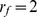 corresponds to hemispherical granule tops. In order to predict contact angles above 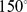 a roughness parameter of over 3 is needed, which is clearly higher than what the SEM images indicate. The Choi model is not able to differentiate between the superhydrophobic and non-superhydrophobic species regardless of what value is assumed for 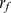. Finally, the assumption that the differential area fraction in the advancing direction 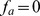 is a result of the Cassie-Baxter (i.e. suspended) wetting state, which is the state modelled by the Choi model. Assuming a non-zero fraction will reduce the predicted advancing contact angles from perfect non-wetting, resulting in two values for the model (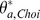 and 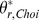), but will not affect the values of 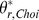 which are the results discussed in this paper. We conclude that the established models can not be used to estimate the wetting properties of Collembola cuticles as the calculated values diverge too much from the measured values. This discrepancy between measured and estimated contact angles might indicate that either the models need to be modified, or that important parameters are not taken into account.

Overhang (reentrant granule geometries) has been suggested as such a parameter [19]. Overhang is not readily included in the classical models as only surface features are taken into account, what is going on beneath the surface in contact with the droplet is not considered at all by the Cassie-Baxter model for example. The presence of overhang would lead to a slightly larger roughness coefficient (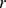) in the Wenzel model, but the increase would be largely insignificant compared to the discrepancy between the predictions of the Wenzel model and the measured contact angles. The role of overhang is largely that of increasing the energy barrier between the Cassie-Baxter state and the Wenzel state, such that it prevents drops from spreading down between the granules on the cuticle surface [34], [35]. This means overhang can readily explain why a drop would stay in the Cassie-Baxter state instead of the Wenzel state, but it cannot explain why the measured contact angles are larger than those predicted by the Cassie-Baxter or Choi models.

Overhang is present on some, but not all of the studied Collembola. Of the two non-superhydrophobic species, *X. maritima* has overhang on the granules, while *C. clavatus* does not. Of the superhydrophobic group several species have overhang (e.g. *A. septentrionalis* and *D. oliviaca*) while several others lack overhang (e.g. *F. quadrioculata* and *A. laricis*). Thus, in contrast to the suggestion of Helbig et al. [19], our results do not indicate a direct link between the presence of overhanging structures and superhydrophobicity. In fact, there is no single parameter that explains why *X. maritima* and *C. clavatus* do not display the same superhydrophobic effect as all the other tested species.

The secondary granules present on some Collembola are significantly larger than the primary granules. This results in larger values for the intergranular distance (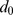) and granule height (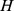) when secondary granules are considered. The effect of secondary granules seem uncertain. In a purely Cassie-Baxter wetting state the presence of secondary granules would completely mask the effect of primary granules as any water would be suspended on the tops of the larger, secondary granules. This results in a significantly lower solid area fraction (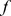) when the secondary granules are considered, and consequently higher estimated contact angles (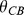). In a Wenzel wetting state the secondary granules would slightly increase the roughness (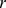) and thus the estimated contact angle (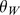). However both models still severely underestimate the contact angle. The presence of secondary granules did not influence the estimated or measured values for the contact angles significantly. Examples of closely related Collembola species from very different humidity conditions showing almost identical secondary granule configurations are included as supporting information, figure S7. The lack of variation in the secondary granules for a wide variation in humidity indicate that the secondary granules are not a key part in the adaptation to humidity conditions.

Collembola normally possess a cover of microscopic hairs. The number of hairs and their arrangement are usually sufficiently conservative to be used as a taxonomic tool [26], but the length of the hairs may vary greatly even between closely related species. Wetting properties are also affected by the number and length of the hairs covering the body surface. [22], [39] If the hair-cover, rather than the cuticle structure, is quantified and used with existing wetting models different values for the contact angles would be predicted. A cover of curved hairs may act in much the same way as structures with overhang and provide robust superhydrophobicity [40]. However, for Collembola the hairs are of microns to tens of microns in scale while the granules are on a scale of hundreds of nanometers, models that incorporate the contact line energy will therefore differ between the two and would be more likely to predict high contact angles for the granules.

Superhydrophobic cuticles were observed for a variety of Collembola species from different families and several different habitats. This was not a universal trait however as two of the tested species did not display the superhydrophobic effect, including a Collembola adapted to extreme drought (*X. maritima*) and one adapted to aquatic habitats (*C. clavatus*). No single structural parameter was observed that could explain the lack of superhydrophobicity in only two of the species. No direct link was found between structural overhang and superhydrophobicity as both structural overhang, and the lack thereof, was observed on both superhydrophobic and non-superhydrophobic cuticles. The most widely used equations underestimated the contact angles of the cuticles. This indicates that more sophisticated models are needed to explain the observed wetting behavior of Collembola cuticles. Closer study of the reasons behind this underestimation may yield interesting results from a biomimetics point of view, as a novel solution for achieving robust superhydrophobicity.

## Materials and Methods

**Figure 7:**
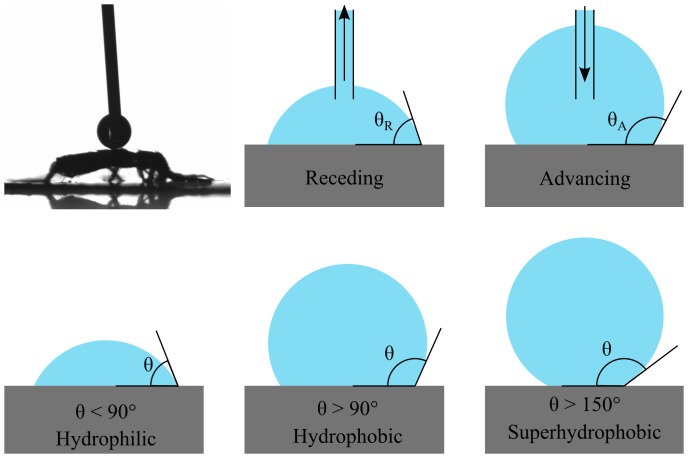
Contact Angles: The contact angle 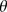 is defined as the angle between the droplet, and the substrate at the contact line. The receding contact angle 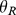 is the contact angle for a droplet with a receding contact line, e.g. for a shrinking droplet, while the advancing contact angle 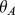 is the contact angle for an advancing contact line, e.g. a growing droplet. Surfaces that when in contact with water display a contact angle of less than 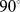 are hydrophilic, surfaces with contact angles of more than 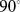 are hydrophobic and surfaces with both advancing and receding contact angles of more than 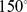 as well as contact angle hysteresis less than 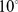 are superhydrophobic. The inset shows a sessile droplet in contact with a springtail.

For droplets in contact with a substrate the contact angle 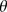 is defined as the angle between the droplet, and the substrate at the contact line (i.e. at the droplet circumference). The receding contact angle 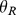 is the contact angle for a droplet with a receding contact line, e.g. for a shrinking droplet, while the advanncing contact angle 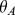 is the contact angle for an advancing contact line, e.g. a growing droplet. The difference between the advancing and receding contact angles is deemed contact angle hysteresis 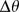. The quantities 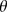, 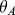, 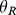 and 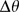 are illustrated in figure 7.

Contact angles were measured with the sessile drop method, using a KSV CAM 200 contact angle goniometer and KSV CAM Optical Contact Angle and Pendant Drop Surface Tension Software v.4.04. The test liquid, de-ionized micropore water, was deposided with a syringe on the dorsal metasoma of Collembola that were fixed to microscope slides with double-sided adhesive tape. Advancing and receding contact angles were attained by leaving the syringe tip in the droplet and adding or siphoning liquid. An example of a droplet in contact with a Collembola is shown in the inset of figure 7. Uncertainties were estimated based on the sample standard deviation of the data set. The samples were classified as superhydrophobic or not superhydrophobic based on the following criteria: both the advancing and receding contact angles (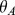 and 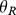) should exceed 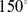 and the contact angle hysteresis (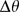) should not exceed 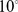.

There are two general models for the interaction between liquids and rough surfaces. The liquid can be in complete contact with the surfaces, filling any openings between asperities. This model is referred to as the Wenzel state [27]. The second model assumes the liquid will not penetrate between the asperities on a rough surface, keeping the liquid suspended in a composite contact partially touching the tops of the asperities and partially hanging suspended between the asperities. This is referred to as the Cassie-Baxter or composite state [28]. Considerable research has been conducted since the initial works of Cassie, Baxter and Wenzel. Modern theories of wetting, such as those of Choi [30] and Dufour [29], consider more complex partial wetting states and differential approaches where simple area fractions are no longer used; surfaces with submicron structures also makes it important to consider the three-phase contact line [29], [30], [41], [42].

The approach of Choi et al.[30] is a variation of the Cassie-Baxter model; suspended wetting is assumed, but instead of using a single value for the solid area fraction (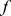) a differential approach is used with different values for the solid area fraction in the advancing and receding directions. Dufour's model [29], is a purely mechanical approach to describing contact angle hysteresis. The deformation energy needed to ''stretch'' drops before they detach from a surface is considered. For a system where the drop rests on the top of asperities, such as the granules of Collembola, the deformed volumes from each asperity corresponds to the solids of revolution of catenary curves. These deformation volumes can be estimated from the size and shape of the asperities. The contact angle hysteresis (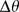) can then be estimated, either from an assumed value for the apparent advancing contact angle, or a posteriori from measured values of the apparent advancing contact angle.

The sizes of the the thinner and thicker parts of the cuticle structures were measured, as well as the height of and distances between these structures. Theses sizes were measured from Scanning Electron Microscopy (SEM) images taken with a Hitachi Su-6600 or with a FEI Helios NanoLab DualBeam FIB (using the electron beam). The samples were freeze dried and fixed to stubs using silver glue or carbon tape. The samples were coated with a thin layer of carbon and sputter coated with Pt.

Focused Ion Beam (FIB) milling was performed with a FEI Helios NanoLab DualBeam FIB to obtain cross-sectional SEM images of the surface structures. The samples were prepared as for general SEM imaging. Subsequently a thick (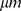 scale) protective layer of Pt was applied using the deposition mode of the instrument. Then a cubic section of the cuticle was removed with the ion beam, the region of removal was placed such that it intersected one or more granules. Afterwards the sample was tilted such that the cross-section of the granules could be imaged.

Collembola are non-regulated invertebrates which are not subject to any special laws or regulations related to animal experiments in Norway. The species studied are not endangered or protected. The samples were identified according to the key of Arne Fjellberg [32], species 3 was only identified to the family of Onychiuridae and not to a specific species within this family. Samples were collected in the field at various locations in southern Norway, except species 3 which was from a lab stock of Azorean origin (held by Leinaas). All field locations were in public areas with no special restrictions on the gathering of invertebrates. The samples were kept at high relative humidity on moist plaster of paris and fed with bark covered in green algae. The samples were killed with chloroform vapor immediately preceding the experiments to ensure freshness.

Twelve species were selected for this investigation. The selection was made to present the different surface structure modifications in Collembola, as well as presenting species from different families and from habitats ranging from extremely dry to very wet. The species included of the order Poduromorpha: *Hypogastura viatica* and *Xenylla maritima* from the family Hypogasturidae and an unidentified species from the Onychiuridae family; and the order Entomobryomorpha: *Anurophorus laricis*, *Anurophorus septentrionalis*, *Folsomia quadrioculata*, *Archisotoma besselsi*, *Cryptopygus clavatus*, *Desoria olivacea*, *Isotomurus prasis* and *Isotoma anglicana* from the family Isotomidae and *Orchesella flavescens* from the family Entomobryidae.

The following information on the species is based on [26], [32] and own observations: *H. viatica* is a surface active species living at, or near, the intertidal zone, it moves away from the water at high tide. *X. maritima*, though of the same family as *H. viatica*, inhabits a very different habitat, it lives on the crusts of lichens on boulders and standing tree trunks, which may beome very dry for prolonged periods; the species is highly drought resistant. *A. laricis* is from the same habitat as and often co-occurs with *X. maritima*. *A. septentrionalis* occurs in moderately dry forest floor, it is closely related to *A. laricis*. *F. quadrioculata* is a species typical for the lower litter layer, a habitat that is rarely flooded but may sometimes become quite wet, it is not surface active and is sensitive to dessication. *A. besselsi* is an intertidal species, which unlike *H. viatica* may become submerged during high tide. *C. clavatus* is found in association with rock pools and other small bodies of water near the shore. It is active under water, where it feeds on algea while submerged without showing signs of plastron formation. *D. olivacea* is a surface dwelling speces from wet habitats with water logged soil. *I. prasis* is a big, surface dwelling species found in wet and humid habitats, though usually not in the same habitats as *D. olivacea*. *I. anglicana* is a big surface dwelling species found in moderately humid habitats both on the forest floor and on open land. *O. flavescens* is another big, surface dwelling speceis that may be found together with *I. anglicana* as well as in wet, marshy habitats.

## Supporting Information

Figure S1 **SEM images of species 1 through 6.** Top left: species 1 *H. viatica* 10 000X magnification, Top right: species 2 *I. prasis* 10 000X magnification, Mid left: species 3 *Onychiurus sp.* 20 000X magnification, Mid right: species 4 *F. quadrioculata* 10 000X magnification, Bottom left: species 5 *A. septentrionalis* 10 000X magnification, Bottom right: species 6 *D. olivacea* 10 000X magnification. The structures shown are typical for the dorsal metasoma.
(TIF)

Figure S2 **SEM images of species 7 through 12.** Top left: species 7 *A. besselsi* 20 000X magnification, Top right: species 8 *C. clavatus* 15 000X magnification, Mid left: species 9 *O. flavescens* 10 000X magnification, Mid right: species 10 *A. laricis* 10 000X magnification, Bottom left: species 11 *I. anglicana* 10 000X magnification, Bottom right: species 12 *X. maritima* 10 000X magnification. The structures shown are typical for the dorsal metasoma.
(TIF)

Figure S3 **Cross section SEM image of species 1 through 6.** Top left: species 1 *H. viatica* 20 004X magnification, overhang is present, Top right: species 2 *I. prasis* 20 000X magnification, no overhang, Mid left: species 3 *Onychiurus sp.* 50 000X magnification, overhang is present Mid right: species 4 *F. quadrioculata* 25 000X magnification, no overhang, Bottom left: species 5 *A. septentrionalis* 20 004X magnification, overhang is present, Bottom right: species 6 *D. olivacea* 50 000X magnification, overhang is present. The structures shown are typical for the dorsal metasoma.
(TIF)

Figure S4 **Cross section SEM image of species 7 through 12.** Top left: species 7 *A. besselsi* 50 000X magnification, overhang is present, Top right: species 8 *C. clavatus* 20 000X magnification, no overhang, Mid left: species 9 *O. flavescens* 35 005X magnification, no overhang, Mid right: species 10 *A. laricis* 8 000X magnification, no overhang, Bottom left: species 11 *I. anglicana* 120 000X magnification, overhang is present, Bottom right: species 12 *X. maritima* 15 000X magnification, overhang is present. The structures shown are typical for the dorsal metasoma.
(TIF)

Figure S5 **Nanoindenter AFM (Ni-AFM) image of species 1 through 6.** Top left: species 1 *H. viatica* Top right: species 2 *I. prasis* Mid left: species 3 *Onychiurus sp.* Mid right: species 4 *F. quadrioculata* Bottom left: species 5 *A. septentrionalis* Bottom right: species 6 *D. olivacea*. The magnifications are indicated by the scale bars. The structures shown are typical for the dorsal metasoma.
(TIF)

Figure S6 **Nanoindenter AFM (Ni-AFM) image of species 7 through 12.** Top left: species 7 *A. besselsi* Top right: species 8 *C. clavatus* Mid left: species 9 *O. flavescens* Mid right: species 11 *I. anglicana* Bottom left: species 12 *X. maritima*. The structures shown are typical for the dorsal metasoma, with the exception of species 9 where the structure of the head is shown, due to challenges in imaging the metasoma.
(TIF)

Figure S7 **Scanning Electron Microscope (SEM) images showing closely related species with secondary granules.** Top left: *H. tullbergi*, Top right: *H. viatica*, Bottom: *C. longispina*. The images have 5000X magnification and show both secondary and primary cuticle granules.
(TIF)

Table S1
**Surface Structure Characteristics Based on NI-AFM**. Surface structure characteristics, as measured on nanoindenter atomic force micrographs. 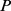: number of edges in the closest equivalent polygon; 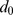: longest regular distance between primary granules; 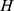: height of granules; L: length of the three-phase contact line for the wetting system of one granule; 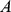: nominal area of a section of cuticle containing a single granule; 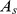 nominal surface area of a granule. Rows marked * present values based on secondary granules.(PDF)

Table S2
**Calculated Parameters Based on NI-AFM**. Calculated parameters, based on nanoindenter atomic force micrographs; 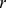: roughness factor, 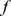: solid area fraction, 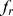: differential solid area fraction, receding direction, 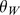: Estimated contact angle from the Wenzel equation, 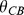: Estimated contact angle from the Cassie-Baxter equation, 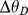: estimated contact angle hysteresis based on Dufour's method and 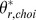: estimated receding contact angle based on Choi's method. Rows marked * present values based on secondary granules.(PDF)
