# Correction: Surface Structure and Wetting Characteristics of Collembola Cuticles

**DOI:** 10.1371/journal.pone.0102961

**Published:** 2014-07-11

**Authors:** The PLOS ONE Staff

The images for Figures 3 and 5 are incorrectly switched. The images for Figures 4 and 6 are also incorrectly switched. The figure legends appear in the correct order. Please find the corrected figures and their legends below.

**Figure 3:**
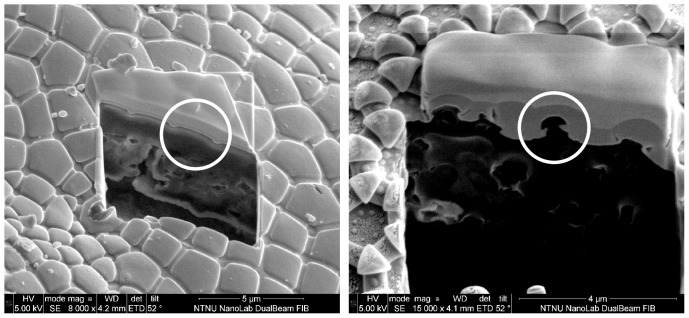
SEM images of FIB cross sections of species 10 and 12. Left: species 10 *A. laricis* magnification 8 000X, Right: species 12 *X. maritima* 15 000X magnification. The images show sections of the cuticle where a prism shaped part has been removed by FIB milling, while the structuring around it was protected by a layer of platinum, to reveal cross sections of the granules. A single granule is highlighted by a white circle in each image. In species 10 there is no evidence of overhang, in species 12 overhang is present.

**Figure 4:**
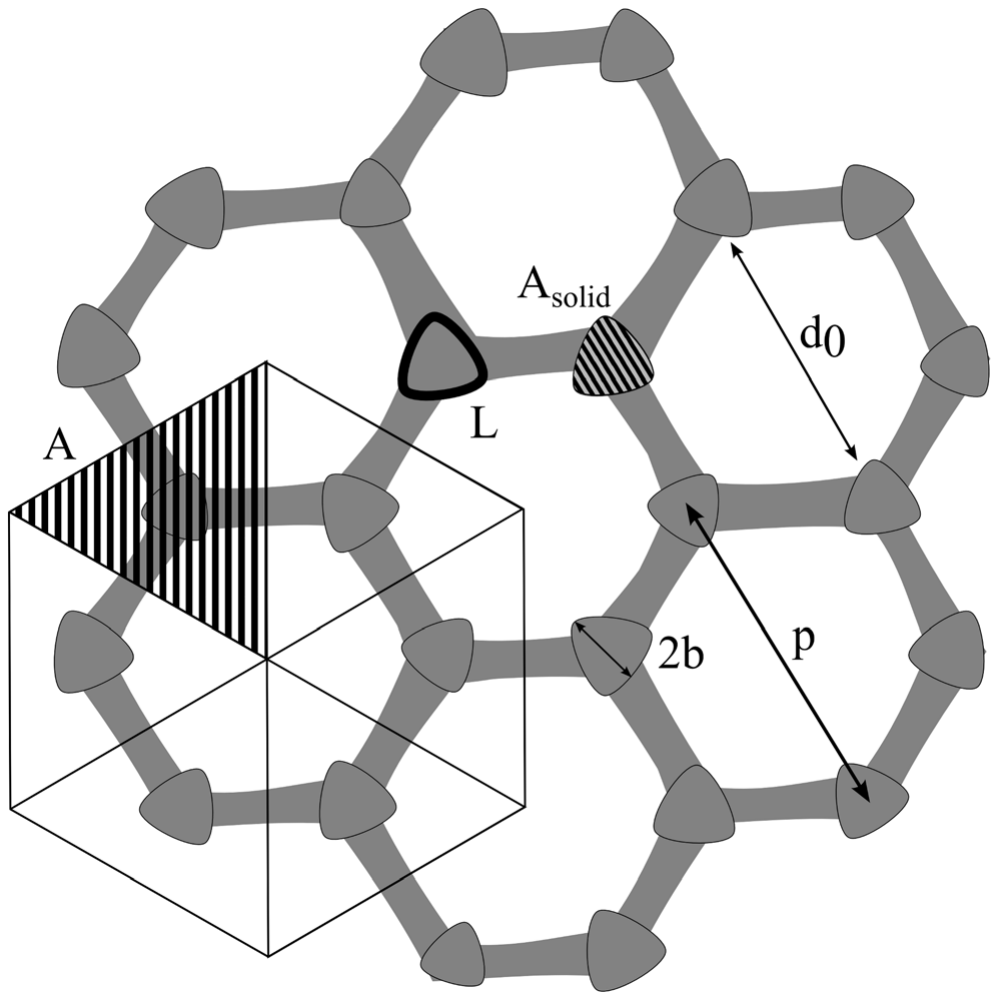
Measurement of structural parameters. The schematic shows how the structural parameters 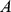, 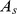, 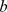, 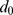, 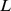 and 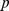 were measured from SEM images.

**Figure 5:**
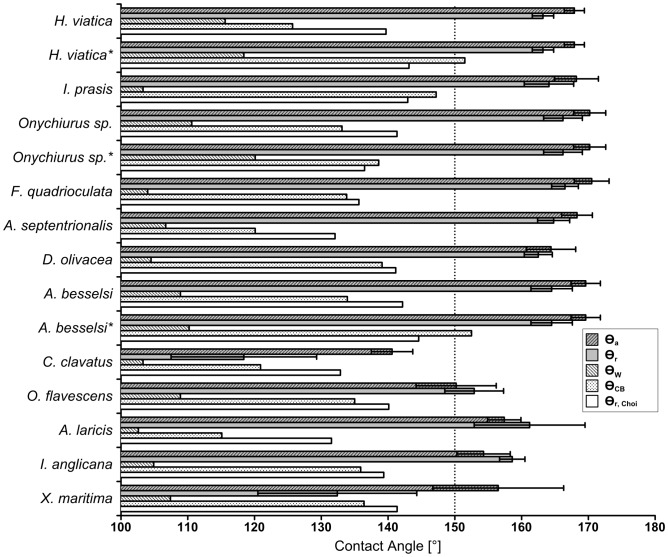
Measured contact angles compared to predicted contact angles. Measured advancing (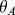) and receding (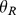) contact angles with one standard deviation error bars as compared to the values predicted by the Wenzel (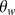), Cassie-Baxter (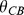) and the Choi (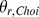) equations. The minimum limit for contact angles considered superhydrophobic is denoted by a dotted line at 150°. Rows marked with an asterisk (*) denote predicted values based on secondary granules.

**Figure 6:**
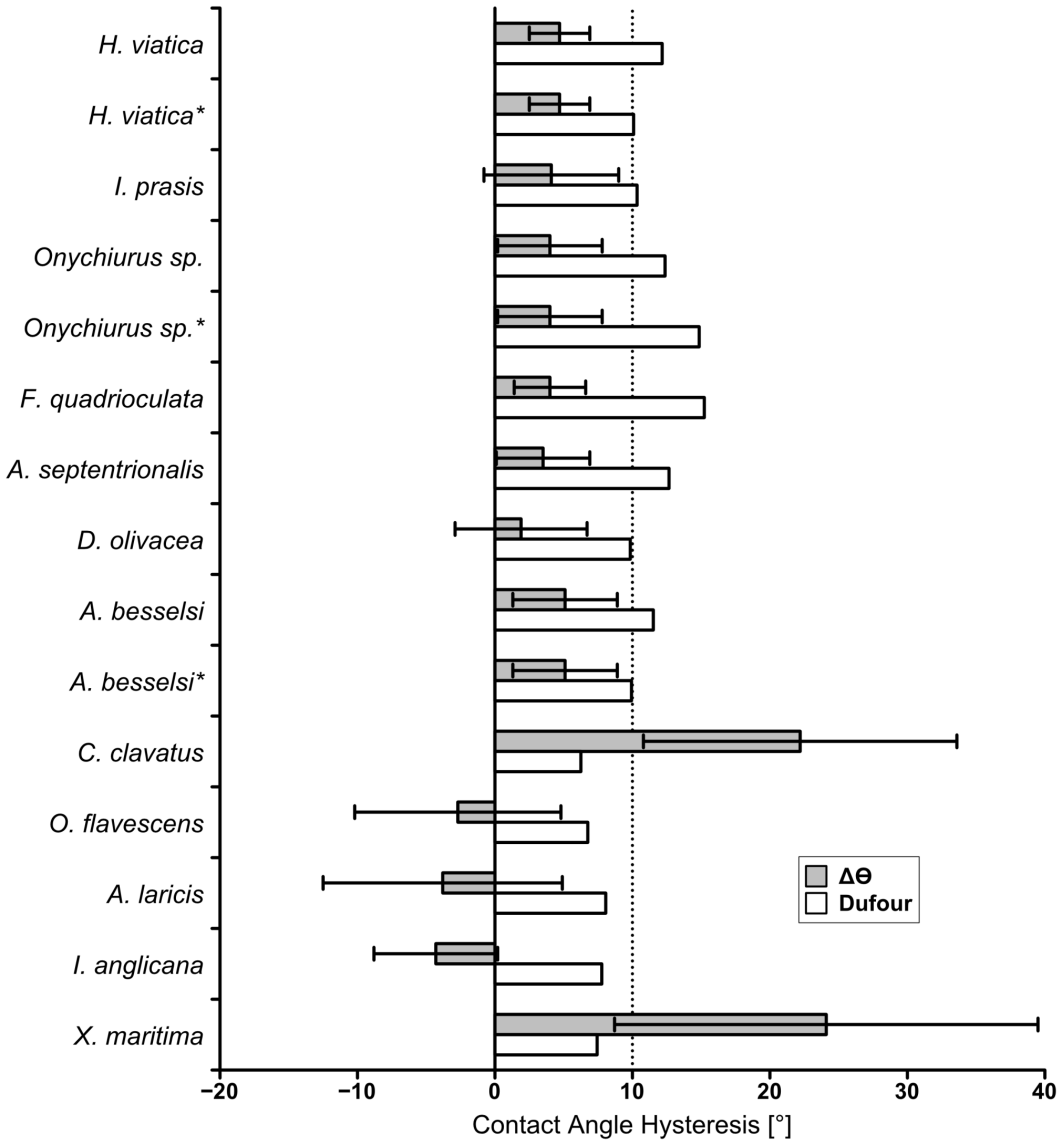
Measured contact angles hysteresis compared to contact angle hysteresis predicted by the Dufour method. The maximum limit for contact angle hysteresis considered superhydrophobic is denoted by a dotted line at 10°. Rows marked with an asterisk (*) denote predicted values based on secondary granules.
