# Supplementary figures and images for: Surface Structure and Wetting Characteristics of Collembola Cuticles
Source: PLoS One. 2014 Feb 3;9(2):e86783. doi: 10.1371/journal.pone.0086783 (PMC3911920; doi:10.1371/journal.pone.0086783)

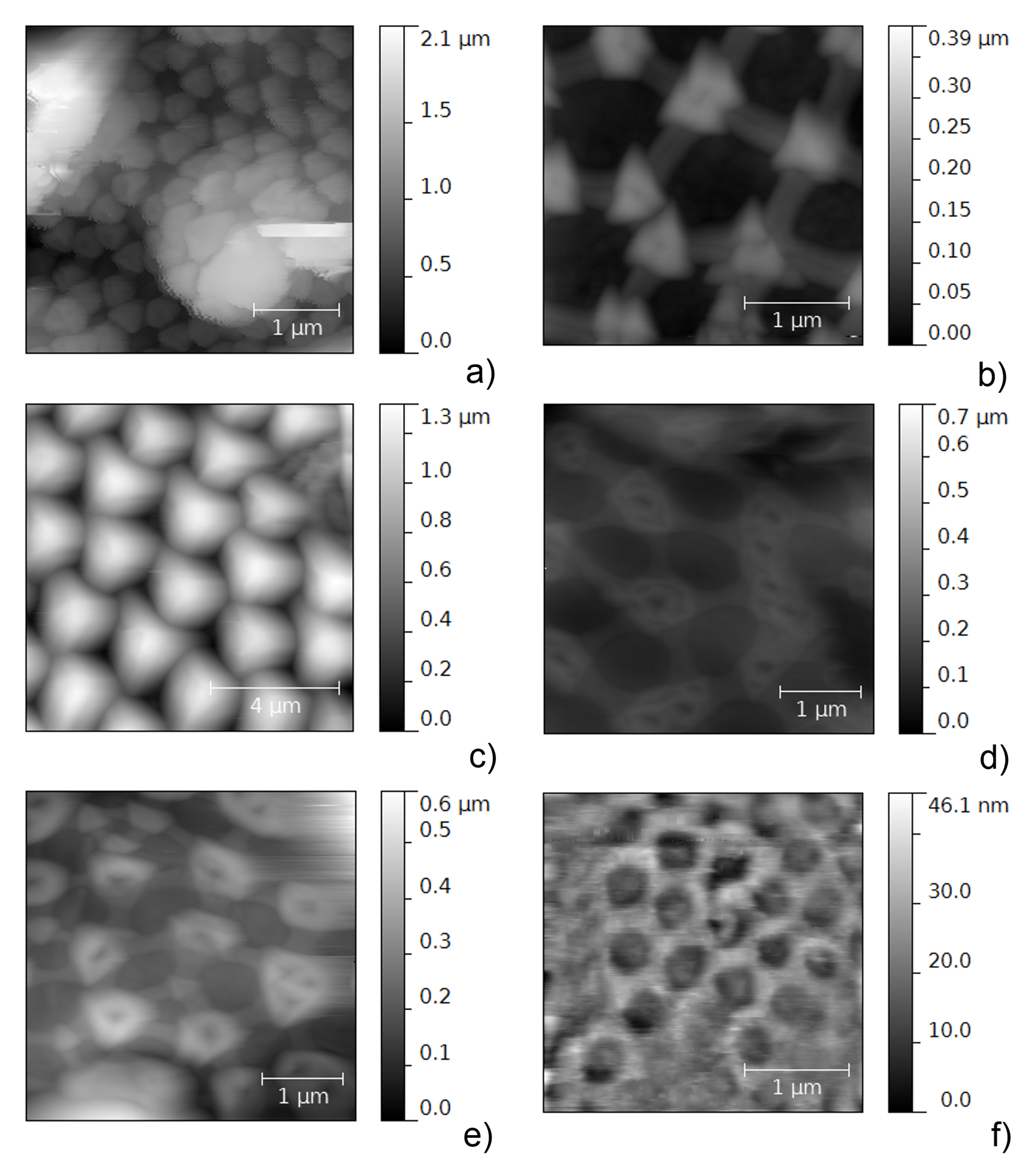

Supplement: Figure S5 — Nanoindenter AFM (Ni-AFM) image of species 1 through 6. Top left: species 1 H. viatica Top right: species 2 I. prasis Mid left: species 3 Onychiurus sp. Mid right: species 4 F. quadrioculata Bottom left: species 5 A. septentrionalis Bottom right: species 6 D. olivacea. The magnifications are indicated by the scale bars. The structures shown are typical for the dorsal metasoma. (TIF) [file pone.0086783.s005.tif]

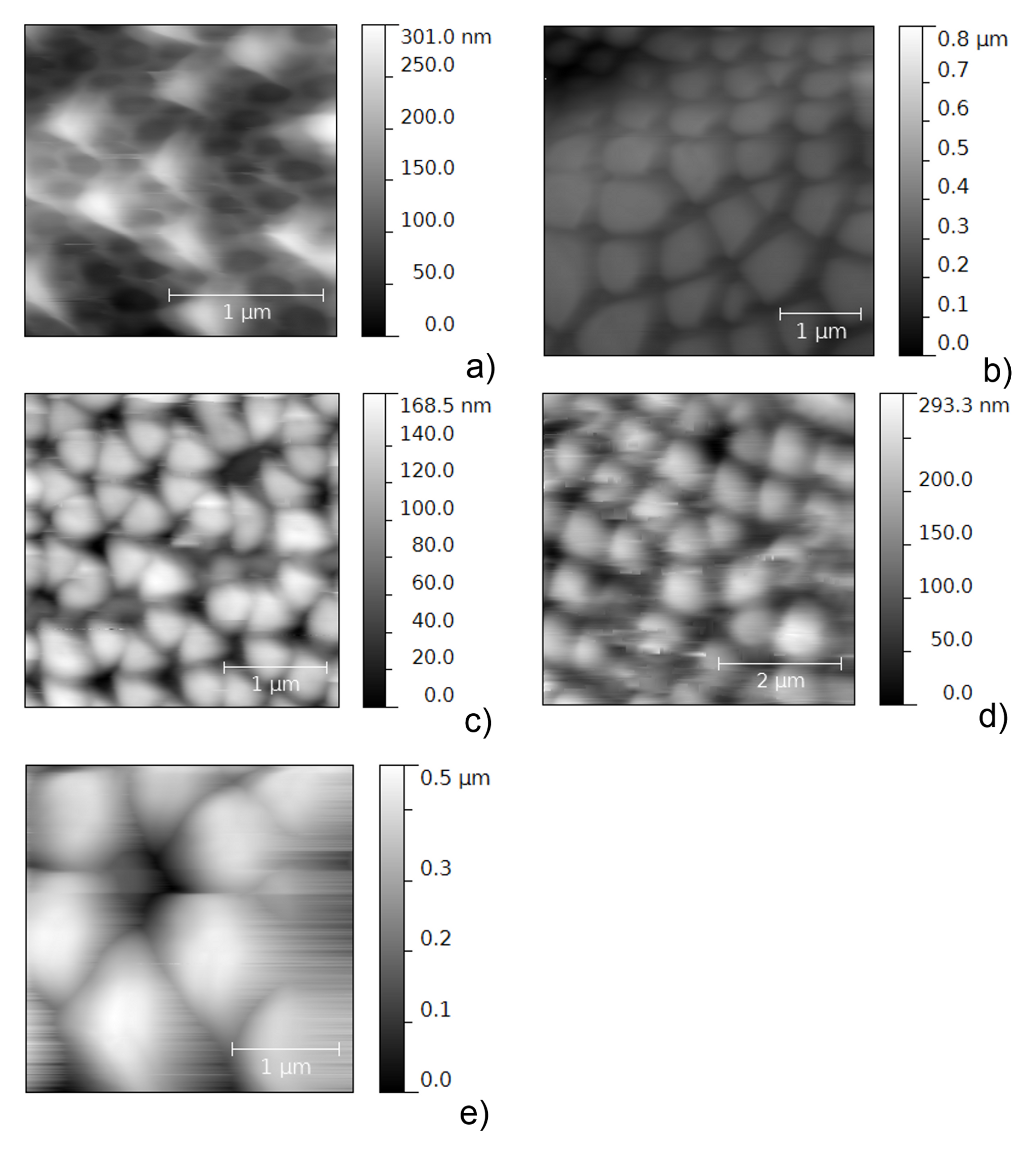

Supplement: Figure S6 — Nanoindenter AFM (Ni-AFM) image of species 7 through 12. Top left: species 7 A. besselsi Top right: species 8 C. clavatus Mid left: species 9 O. flavescens Mid right: species 11 I. anglicana Bottom left: species 12 X. maritima. The structures shown are typical for the dorsal metasoma, with the exception of species 9 where the structure of the head is shown, due to challenges in imaging the metasoma. (TIF) [file pone.0086783.s006.tif]

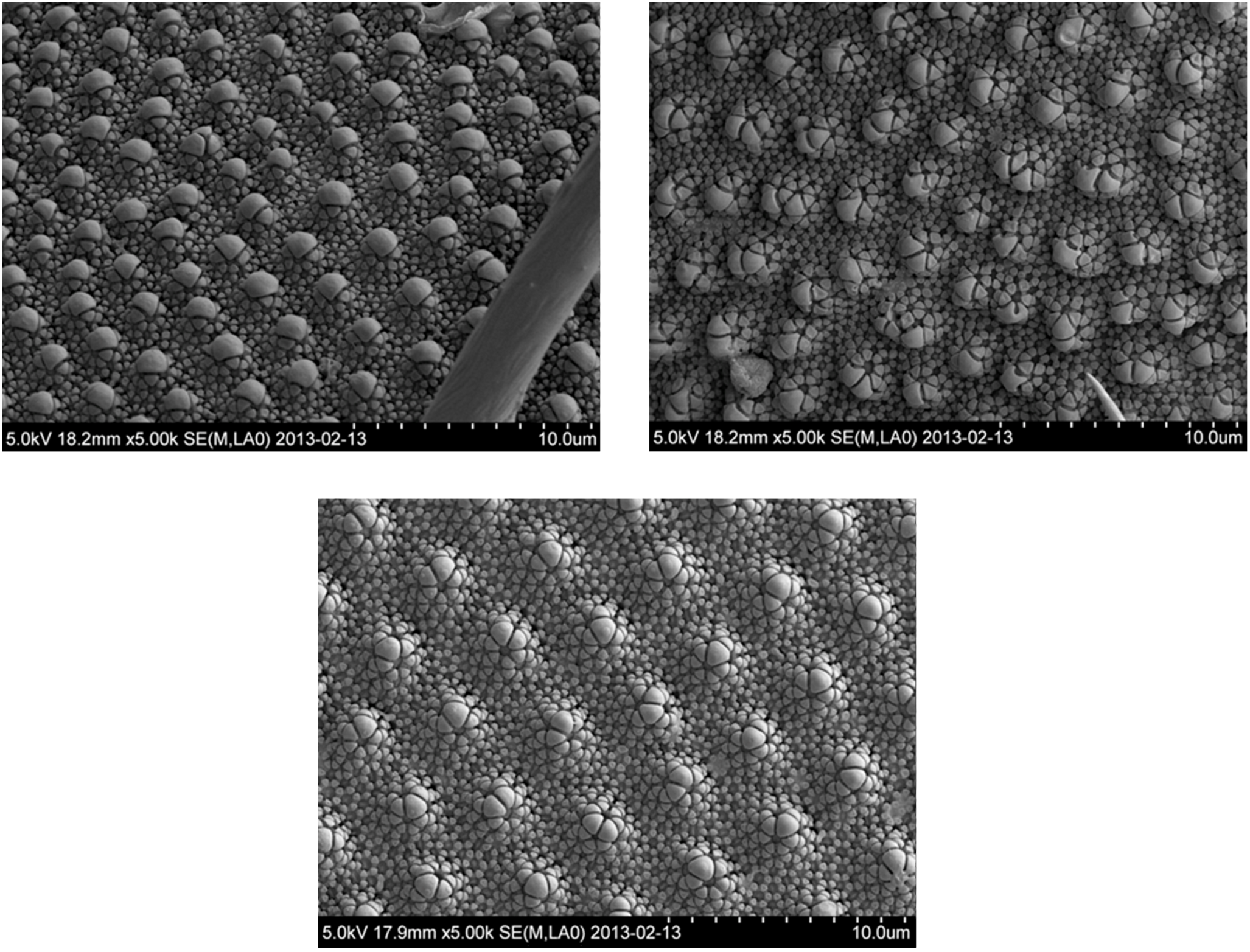

Supplement: Figure S7 — Scanning Electron Microscope (SEM) images showing closely related species with secondary granules. Top left: H. tullbergi, Top right: H. viatica, Bottom: C. longispina. The images have 5000X magnification and show both secondary and primary cuticle granules. (TIF) [file pone.0086783.s007.tif]
