# Supplementary material for: Surface Structure and Wetting Characteristics of Collembola Cuticles
Source: PLoS One. 2014 Feb 3;9(2):e86783. doi: 10.1371/journal.pone.0086783 (PMC3911920; doi:10.1371/journal.pone.0086783)
Supplement: Table S1 — Surface Structure Characteristics Based on NI-AFM. Surface structure characteristics, as measured on nanoindenter atomic force micrographs. : number of edges in the closest equivalent polygon; : longest regular distance between primary granules; : height of granules; L: length of the three-phase contact line for the wetting system of one granule; : nominal area of a section of cuticle containing a single granule; nominal surface area of a granule. Rows marked * present values based on secondary granules. (PDF) [file pone.0086783.s008.pdf]

#	Species	P	d_0 [nm]	H [nm]	L [nm]	A [μm^2]	A_s [μm^2]
1	*H. viatica*	6	310	310	1210	0.15	0.10
1*	*H. viatica*	3	2110	1500	3640	8.00	0.99
2	*I. prasis*	5	790	150	1300	0.60	0.13
3*	*Onychiurus*	3	1490	950	4560	8.85	1.51
4	*F. quadrioculata*	4	730	120	2400	0.93	0.40
5	*A. septentrionalis*	3	540	150	1990	0.73	0.29
6	*D. oliviaca*	6	360	180	950	0.14	0.058
7	*A. besselsi*	6	230	40	480	0.35	0.017
7*	*A. besselsi*	4	520	140	1420	0.57	0.14
8	*C. clavatus*	4	300	130	1750	0.42	0.22
9	*O. flavescens*	6	230	120	1410	0.19	0.14
11	*I. anglicana*	4	590	180	2000	0.64	0.30
12	*X. maritima*	6	560	220	2800	0.66	0.55
