# Supplementary material for: Surface Structure and Wetting Characteristics of Collembola Cuticles
Source: PLoS One. 2014 Feb 3;9(2):e86783. doi: 10.1371/journal.pone.0086783 (PMC3911920; doi:10.1371/journal.pone.0086783)
Supplement: Table S2 — Calculated Parameters Based on NI-AFM. Calculated parameters, based on nanoindenter atomic force micrographs; : roughness factor, : solid area fraction, : differential solid area fraction, receding direction, : Estimated contact angle from the Wenzel equation, : Estimated contact angle from the Cassie-Baxter equation, : estimated contact angle hysteresis based on Dufour's method and : estimated receding contact angle based on Choi's method. Rows marked * present values based on secondary granules. (PDF) [file pone.0086783.s009.pdf]

#	Species	r	f	f_r	θ_W	θ_{CB}	$\Delta\theta_D$	$\theta^*_{r,Choi}$
1	*H. viatica*	2.64	0.66	0.33	117.3°	116.8°	13.6°	136.4°
1*	*H. viatica*	3.07	0.12	0.23	122.2°	153.9°	9.6°	143.9°
2	*I. prasis*	1.28	0.22	0.21	102.9°	144.9°	8.8°	145.4°
3*	*Onychiurus*	2.13	0.17	0.31	111.7°	149.2°	14.1°	138.3°
4	*F. quadrioculata*	1.24	0.44	0.33	102.5°	129.7°	14.9°	136.8°
5	*A. septentrionalis*	1.45	0.40	0.34	104.6°	131.8°	14.0°	135.8°
6	*D. oliviaca*	1.62	0.43	0.27	106.4°	130.4°	9.7°	141.3°
7	*A. besselsi*	1.54	0.049	0.27	105.6°	163.7°	12.3°	141.0°
7*	*A. besselsi*	1.82	0.24	0.28	108.4°	143.0°	12.5°	140.5°
8	*C. clavatus*	1.46	0.53	0.36	104.7°	124.5°	6.6°	134.7°
9	*O. flavescens*	1.28	0.71	0.38	102.3°	114.3°	7.9°	133.6°
11	*I. anglicana*	1.53	0.47	0.33	105.5°	127.6°	8.6°	136.9°
12	*X. maritima*	1.54	0.83	0.35	105.5°	108.1°	9.5°	135.1°
